# *Methanosphaera stadtmanae* induces a type IV hypersensitivity response in a mouse model of airway inflammation

**DOI:** 10.14814/phy2.13163

**Published:** 2017-03-31

**Authors:** Emilie Bernatchez, Matthew J. Gold, Anick Langlois, Pascale Blais‐Lecours, Magali Boucher, Caroline Duchaine, David Marsolais, Kelly M. McNagny, Marie‐Renée Blanchet

**Affiliations:** ^1^ Institut Universitaire de Cardiologie et de Pneumologie de QuébecUniversité LavalQuebec CityQuebecCanada; ^2^ The Biomedical Research CenterUniversity of British ColumbiaVancouverBritish ColumbiaCanada

**Keywords:** Eosinophils, hypersensitivity response, IL‐17, lung, methanogens

## Abstract

Despite improved awareness of work‐related diseases and preventive measures, many workers are still at high risk of developing occupational hypersensitivity airway diseases. This stems from a lack of knowledge of bioaerosol composition and their potential effects on human health. Recently, archaea species were identified in bioaerosols, raising the possibility that they play a major role in exposure‐related pathology. Specifically, *Methanosphaera stadtmanae* (MSS) and *Methanobrevibacter smithii* (MBS) are found in high concentrations in agricultural environments and respiratory exposure to crude extract demonstrates immunomodulatory activity in mice. Nevertheless, our knowledge of the specific impact of methanogens exposure on airway immunity and their potential to induce airway hypersensitivity responses in workers remains scant. Analysis of the lung mucosal response to methanogen crude extracts in mice demonstrated that MSS and MBS predominantly induced T_H_17 airway inflammation, typical of a type IV hypersensitivity response. Furthermore, the response to MSS was associated with antigen‐specific IgG_1_ and IgG_2a_ production. However, despite the presence of eosinophils after MSS exposure, only a weak T_H_2 response and no airway hyperresponsiveness were observed. Finally, using eosinophil and mast cell‐deficient mice, we confirmed that these cells are dispensable for the T_H_17 response to MSS, although eosinophils likely contribute to the exacerbation of inflammatory processes induced by MSS crude extract exposure. We conclude that, as MSS induces a clear type IV hypersensitivity lung response, it has the potential to be harmful to workers frequently exposed to this methanogen, and that preventive measures should be taken to avoid chronic hypersensitivity disease development in workers.

## Background

To date, despite treatments and avoidance/prevention measures (Jolly et al. 2015; Cano‐Jimenez et al. 2016; Suojalehto et al. 2016), workers in various environments are still regularly diagnosed with occupational lung hypersensitivity diseases such as asthma, obstructive pulmonary disease and hypersensitivity pneumonitis (HP) (Baldassarre et al. 2016; Burge 2016; Cushen et al. 2016; Gao and Li 2016; Kraim‐Leleu et al. 2016). The struggle in preventing these occupational diseases stems from the presence of unknown antigens, including microorganisms, in environmental bioaerosols and their unknown potential to elicit an inflammatory response in the lung. A better understanding of the nature and immunogenicity of these antigens would lead to improved prevention and a better quality of life for workers.

Interestingly, non‐viable microorganisms from the archaea domain, namely methanogens from the *Methanobacteriacaea* group (*Methanobrevibacter* or *Methanosphaera* genus), were found in high concentrations (up to 10^8^ archaea/m^3^) in bioaerosols from poultries, dairy farms and swine confinement buildings (Nehme et al. 2009; Blais‐Lecours et al. 2012; Just et al. 2013). Furthermore, a mouse model of lung inflammation induced by crude extracts of these methanogens was recently developed and demonstrated species‐dependent lung immune responses to *Methanosphaera stadtmanae* (MSS) and *Methanobrevibacter smithii* (MBS), with MSS being the more potent inducer (Blais‐Lecours et al. 2011). This study demonstrated that archaeal crude extracts induce the recruitment of CD4^+^ and CD19^+^ cells in the lung along with a strong production of serum IgG (Blais‐Lecours et al. 2011). Importantly, human endogenous viable methanogens species are associated with oral diseases (Lepp et al. 2004; Vianna et al. 2006, 2008, 2009; Vickerman et al. 2007; Jiang et al. 2009; Efenberger et al. 2015), intestinal diseases (Scanlan et al. 2008; Lee et al. 2013; Blais‐Lecours et al. 2014; Mira‐Pascual et al. 2015) and obesity (Zhang et al. 2009; Mbakwa et al. 2015). Methanogens activate human peripheral blood cells to release the important immune mediator TNF (Blais‐Lecours et al. 2014), and methanogen‐specific IgGs are detectable in periodontic and inflammatory bowel disease (IBD) patients, documenting their potential as activators of the human immune system in environments where the strict methanogen conditions allow their survival. Nevertheless, because of a lack of detailed information on the specific immune mechanisms induced by these microorganisms (alive or dead), the role of methanogen‐laden bioaerosols in human inflammatory responses remains unclear.

Hypersensitivity responses are defined as a pathogenic immune response to non‐harmful antigens, and can lead to the development of various occupational hypersensitivity diseases such as occupational asthma and HP. These responses are classically separated in four types. The type 1 hypersensitivity response, also known as the allergic response, is, for example, involved in allergic asthma (Bogaert et al. 2009). It is mainly characterized by the recruitment and activation of eosinophils and mast cells through release of cytokines, such as IL‐4, 5, 13, 33, and eotaxins, by type 2 innate lymphoid cells (ILC2s) and CD4^+^ T cells (T_H_2 CD4^+^ cells) (Hammad and Lambrecht 2015). These also drive isotype switching of B cells and the production of IgE and IgG_1_ immunoglobulins (Snapper et al. 1988). In the lung, chronic activation of this pathway normally leads to the development of airway hyperresponsiveness (AHR) (Lauzon and Martin 2016). Type II and III hypersensitivity responses (the latter being involved in HP (Bogaert et al. 2009)), lead to antibody production (IgG) resulting in either the killing of host cells by induction of apoptosis (type II) or in the formation of precipitates that drive a strong local immune response and tissue injury (type III) (Descotes and Choquet‐Kastylevsky 2001; Rajan 2003; Warrington et al. 2011). Finally, type IV hypersensitivity responses can be found in diseases such as HP (Bogaert et al. 2009). This response is mainly cell‐mediated, either by secretion of inflammatory mediators by type 1 (T_H_1) and type 17 (T_H_17) effector CD4^+^ T cells (interferon‐gamma; IFN*γ* by T_H_1, and IL‐17A by T_H_17 cells) or by the cytotoxic activity of CD8^+^ T cells (Descotes and Choquet‐Kastylevsky 2001; Rajan 2003; Warrington et al. 2011).

Using a mouse model of lung exposure to archaeal crude extracts and an array of genetically modified mice, we set out to characterize the type of hypersensitivity response induced by methanogen exposure to resolve their potential to induce hypersensitivity disease in highly exposed workers (Nehme et al. 2009; Blais‐Lecours et al. 2012; Just et al. 2013). We demonstrate that MSS crude extracts induce a strong eosinophilic response at low dose that is associated with a mixed T_H_2/T_H_17 and a IgG_1_ lung mucosal response, while exposure to high doses result solely in a T_H_17 and a strong IgG_1_/IgG_2a_ response typical of a type IV hypersensitivity response. Furthermore, although eosinophils are present in high quantity and contribute to the inflammatory response to MSS, we show they are dispensable for the development of the type IV hypersensitivity response and, accordingly, that MSS fails to induce AHR. We find that MBS crude extract also induces a T_H_17 lung response. We conclude that airborne, non‐infectious exposure to methanogen bioaerosols, especially MSS, initiates a type IV hypersensitivity airway response, and that exposure of workers to these bioaerosols should be limited, if not prevented.

## Methods

### Animals

C57BL/6J and C.129S1(B6)‐*Gata1* ^*tm6Sho*^/J (ΔdblGATA) were obtained from Jackson Laboratories and Cpa3‐Cre mice were kindly provided by Feyerabend et al. (2011). Mice were kept in a pathogen‐free animal unit (BRC, University of British Columbia, Vancouver, BC, Canada; CRIUCPQ, Laval University, Québec, QC, Canada) for the duration of the experiments.

### Ethics statement

Experiments were approved by local ethics committees and followed Canadian Animal Care guidelines for the use of experimental mice. The study was approved by the Laval University Committee for Animal Care (protocol 2013‐124‐2). Mice were euthanized by an overdose of ketamine/xylazine according to the committee guidelines on rodent euthanasia.

### Cell culture and preparation of archaeal crude extract

*Methanosphaera stadtmanae* (DSMZ‐11975; MSS) and *Methanobrevibacter smithii* (DSMZ‐3091, MBS) (DSMZ, Germany) were grown according to DSMZ's cultivation conditions. Cells were lyophilized and reconstituted as described by Blais‐Lecours et al. (2011). Endotoxins were quantified in MSS and MBS crude extracts, revealing 124 and 22 EU/mg, respectively, using the Kinetic Chromogenic LAL Assay according to the manufacturer's instructions (Lonza Walkersville, Inc., Walkersville, MD).

### Induction and assessment of the airway inflammation

**Figure 1:**
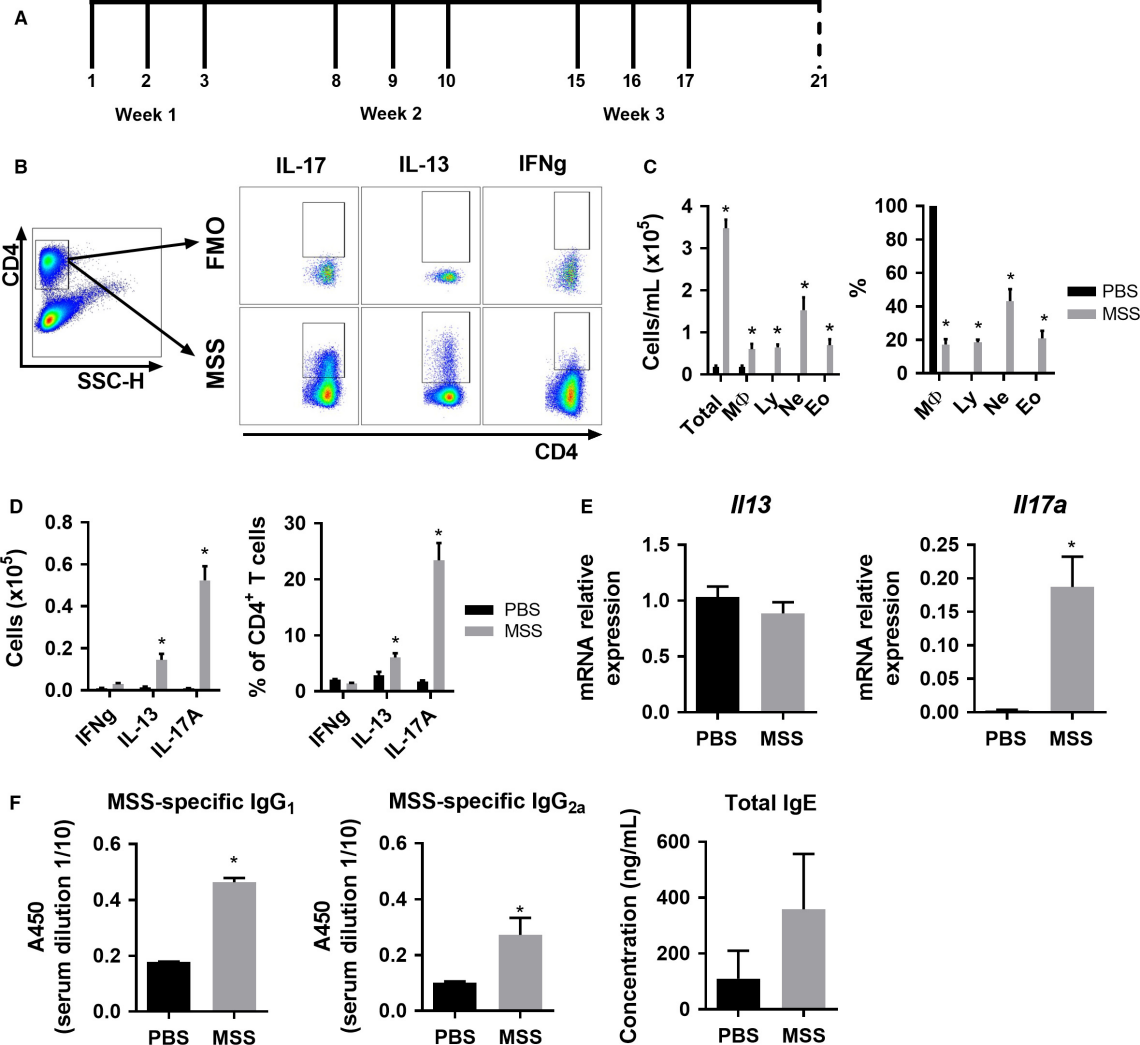
MSS induces a mixed T_H_2/T_H_17 immune lung response. (A) Timeline of the model of exposure to methanogens. Full line represents intranasal instillation of either PBS, MSS or MBS crude extract while dashed line represents day of euthanasia. (B) Flow cytometry gating strategy for the polarity of the effector lung response after ex‐vivo stimulation of lung leukocytes isolated from methanogen‐exposed mice. CD4^+^ T cells were gated from total lung cells and cytokine‐positive cells were analyzed using Fluorescence Minus One (FMO) controls. (C) Severity of the inflammatory lung response after 3 *μ*g MSS exposure was measured using total broncho‐alveolar lavage (BAL) count and differential count. (D) Polarity of the effector response evaluated as number and the % of CD4^+^ cells expressing IFNg, IL‐13 or IL‐17A. (E) *Il13* and *Il17a* expression measured by qRT‐PCR on lung tissue of mice exposed to MSS compared with PBS. (F) MSS‐specific IgG_1_ and IgG_2a_ and total IgE production was measured from serum using ELISA. Results are representative of at least three separate experiments; *n* = 3–6 mice/group. * = *P* ˂ 0.05.

The timeline used for the chronic model is presented in Figure 1A. Mice were anesthetized with isoflurane and received intranasal instillations (i.n.) of either 3 *μ*g or 100 *μ*g of MSS crude extract or 6.25 *μ*g of MBS crude extract on three consecutive days/week for 3 weeks. Mice were euthanized 4 days after the last exposure. Upon euthanasia, mice were tracheotomized with an 18G catheter, and a broncho‐alveolar lavage (BAL) was performed via three separate injections/aspirations of 1 mL saline. Total BAL cells were counted and differential counts obtained using Giemsa stain (HemaStain Set, Fisher Scientific, Kalamazoo, MI). For the administration of anti‐IL‐17A, mice received 50 *μ*g of anti‐IL‐17A or isotype control antibodies (Bio X Cell, West Lebanon, NH) intra‐peritoneally (i.p.) 1 h prior to each MSS i.n. exposure.

### Assessment of the airway hyperresponsiveness

Four days after the final challenge, WT mice were anesthetized with ketamine/xylazine***,*** tracheotomized and intubated with an 18G catheter. The airway resistance (Rrs) was measured with a *Flexivent* apparatus (SCIREQ, Montreal, Qc, Canada). Respiratory frequency was set at 150 breaths/min with a tidal volume of 0.2 mL, and a positive end‐expiratory pressure of 3 mL H_2_O was applied. 50uL of increasing concentrations of methacholine (MCh) (0–64 mg/mL) was administered by nebulization.

### Detection of antigen‐specific IgG_1_ and IgG_2a_ and total IgE

Upon euthanasia, blood was harvested through a cardiac puncture and serum was collected. For IgGs, ELISA plates were coated with 50 *μ*g of MSS, blocked with 10% FBS in PBS and incubated with serum dilutions. Samples were then incubated with either anti‐mouse IgG_1_‐HRP or IgG_2a_‐HRP (BD Biosciences, San Diego, CA). Total IgE were measured using a Mouse IgE ELISA Set (BD OptEIA) according to the manufacturer's instructions. The reactions were revealed with BD OptEIA TMB (BD Biosciences) and stopped with 1N HCl.

### Isolation of lung leukocytes

Lung leukocytes were obtained by digestion of lung tissue with Collagenase IV (Sigma, Oakville, ON, Canada) for 0.5 h at 37°C. Digested tissue was pressed through a 70 *μ*m cell strainer and leukocytes were enriched using a 30% Percoll gradient (GE Healthcare, Uppsala, Sweden). Red blood cells were lysed using ammonium chloride.

### T cell cytokine production assays

CD4^+^ T cell cytokine production was induced by stimulating 1 × 10^6^ lung‐isolated leukocytes with PMA (50 ng/mL), ionomycin (750 ng/mL) and brefeldin A (3 *μ*g/mL) in RPMI 1640 supplemented with 10% FBS and 1% antibiotic/antimycotic in a 96‐well plate for 4 h. Intracellular cytokine production was analyzed by flow cytometry as described below.

### Flow cytometry analysis of isolated leukocytes

Cells were suspended in PBS supplemented with 10% FBS and 0.02% sodium azide for cell surface staining. Cells were stained with CD4 (eBioscience). For intracellular staining, cells were fixed and permeabilized using the Intracellular Fixation & Permeabilization Buffer Set (eBioscience). Antibodies used were: IL‐13, IL‐17A and IFNg (BioLegend). We measured the polarity of the inflammatory response by gating on CD4^+^ T cells from whole lung cells and using fluorescence minus one (FMO) controls (gating presented on Fig. 1B). Lung ILC2s were gated as described in (Gold et al. 2014) and stained with: CD25, ST2, Sca‐1 (eBioscience), CD3e, CD11b, CD11c, CD19, Gr1, NK1.1, Ter119, CD45, and CD90.2 (Ablab, Vancouver, B.C., Canada).

### RNA isolation and quantitative PCR

Lung tissue was homogenized in Trizol and RNA samples were prepared using EZ‐10 DNAaway RNA Mini‐Preps kit (BioBasic Canana INC., Markham, CA). Using iScript cDNA Synthesis Kit (Bio‐rad, Mississauga, CA), cDNA was obtained and used for quantitative RT‐PCR using SsoAdvenced Universal SYBR Green Supermix (Bio‐rad) and a rotor gene 6000 (QIAGEN, Valencia, Calif). Validated primers (efficiency between 90% and 105%) used for quantitative RT‐PCR: *Ccl11 5′‐GAATCACCAACAACAGATGCAC‐3′* (fwr) and *5′‐ATCCTGGACCCACTTCTTCTT‐3′* (rev); *Ccl24 5′‐ATTCTGTGACCATCCCCTCAT‐3′* (fwr) and *5′‐TGTATGTGCCTCTGAACCCAC‐3′* (rev); *Il13 5′‐CCTGGCTCTTGCTTGCCT T‐3′* (fwr) and *5′‐GGTCTTGTGTGATGTTGCTCA‐3′* (rev); *Il17a 5′‐AGCAGCGATCATCCCTCAAAG‐3′* (fwr) and *5′‐TCACAGAGGATATCTATCAGGGTC‐3′* (rev); and *Il33 5′‐GGGAAGAAGGTGATGGTGAA‐3′* (fwr) and *5′‐CCGAAGGACTTTTTGTGAAGG‐3′* (rev). Relative quantification was calculated with 2^−ΔCT^ and using GAPDH and GNB as reference genes (Pfaffl 2001; Vandesompele et al. 2002).

### Statistics

Data are presented as mean ± SEM. Data were tested for normality and homogeneity of variance. When appropriate, variables were log‐transformed. Statistical analysis for multiple comparisons was performed using an ANOVA table followed by Tukey's multiple comparison test or by a Kruskal‐Wallis rank sum test followed by Nemenyi‐Tests. Non‐multiple comparisons were analyzed using unpaired T‐tests. Statistical significance was determined at *P* < 0.05.

## Results

To characterize the immune response induced by MSS and its potential to induce hypersensitivity responses, we first evaluated the polarity of the effector response following exposure to MSS crude extract. MSS crude extract quantities used in our protocol were similar to the ones used by Blais‐Lecours et al. (2011), which are based on the well‐described model of hypersensitivity pneumonitis to *Saccharopolyspora rectivirgula* (SR) antigen and widely accepted to mimic human pathology onset, severity and inflammatory processes (Denis et al. 1991; Gudmundsson and Hunninghake 1997; Nance et al. 2004). To insure the response to MSS in our study was similar to what was previously described (Blais‐Lecours et al. 2011), the broncho‐alveolar lavage (BAL) inflammatory response was analyzed. As described by Blais‐Lecours et al., exposure to low dose of MSS (3 *μ*g) increased the number of immune cells in the BAL (Fig. 1C), and these were characterized by the presence of lymphocytes, neutrophils and eosinophils (Fig. 1C). To complement the analysis previously published by Blais‐Lecours et al., the polarity of the response to MSS was thoroughly studied. The analysis of cytokine polarity production by CD4^+^ T cells revealed a predominant T_H_17 response, as determined by the high frequency of CD4/IL‐17A^+^ cells (Fig. 1D). Interestingly, exposure to 3 *μ*g of MSS also induced weak T_H_2 responses, as denoted by an increased number and % of the CD4/IL‐13^+^ cells (Fig. 1D). Polarization towards T_H_17 was further confirmed by increased expression of *Il17a,* but not *Il13,* via qRT‐PCR (Fig. 1E). Analyses of serum immunoglobulins revealed a strong production of MSS‐specific IgG_1_, a small production of IgG_2a,_ and no IgE production (Fig. 1F). These data suggest that a type IV hypersensitivity response is induced after exposure to low dose of MSS, although we also observed a low level of allergic, type I hypersensitivity response.

**Figure 2:**
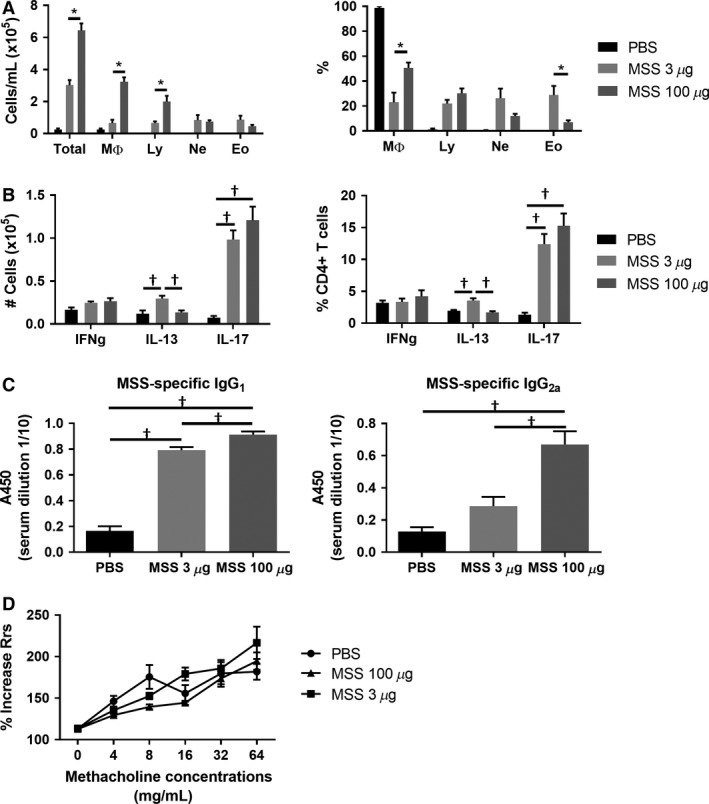
MSS mainly induces a T_H_17 polarized immune response. (A) Severity of the inflammatory response following exposure to 3 *μ*g versus 100 *μ*g of MSS was quantified using total broncho‐alveolar lavage (BAL) count and differential count. Results are representative of at least three separate experiments; *n* = 3–6 mice/group. * = *P* ˂ 0.05. (B) Polarity of the effector response evaluated as number and the % of CD4^+^ cells expressing IFNg, IL‐13 or IL‐17A. (C) MSS‐specific IgG_1_ and IgG_2a_ production was measured from serum using ELISA. Results were pooled from two experiments; *n* = 6–10 mice/group. † = *P* ˂ 0.05 with multi‐comparison test. Using the Flexivent Apparatus, (D) airway resistance (Rrs) was evaluated in mice exposed to 3 *μ*g or 100 *μ*g MSS. Results were pooled from two experiments; *n* = 6–12 mice/group. * = *P* ˂ 0.05.

Similar to previous studies (Blais‐Lecours et al. 2015), an exacerbated total response was observed at 100 *μ*g MSS compared with 3 *μ*g, characterized mainly by macrophages and lymphocytes (Fig. 2A). Of important note is the inversely proportional presence of eosinophils to the quantity of instilled MSS, as denoted by the higher % of eosinophils response at 3 *μ*g MSS compared with 100 *μ*g. This is very interesting as the total number of cells is similar after exposure to low or high quantities of MSS (Fig. 2A). This suggests that different types of hypersensitivity responses can be developed with different levels of MSS exposure, or that the higher dose shifts the timing of the response to include more macrophages at the time of euthanasia. Independently of the explanation, this is an important observation and a significant factor in determining the potential impact of MSS bioaerosols exposure in humans. Additionally, when looking at the effector response, we found that the cell number and the % of CD4/IL‐13^+^ T cells are decreased in mice exposed to 100 *μ*g compared with 3 *μ*g, while the CD4/IL‐17A^+^ T cells population remains unchanged (Fig. 2B). Furthermore, IgG_1_ and IgG_2a_ levels are increased in mice exposed to 100 *μ*g MSS (Fig. 2C), while the IgG_2a_ production in response to 3 *μ*g MSS was very weak. Thus, MSS exposure mainly promotes a T_H_17‐dominated and strong IgG_1_/IgG_2a_ response, indicative of a type IV hypersensitivity response.

As mentioned, the lung T_H_2 response and the presence of eosinophils in BAL are associated with the development of AHR (Gauvreau et al. 1999; Wynn 2015). Using a Flexivent apparatus, we set out to verify whether exposure to MSS crude extract induces an exaggerated increase in the airway resistance (Rrs), which is a measure of AHR, compared with the negative control PBS (Blanchet et al. 2007; Bernatchez et al. 2015). Our group has extensively described that an increase in Rrs in the area of 500% over baseline is considered a positive response for AHR in C57Bl/6J background (Blanchet et al. 2007; Bernatchez et al. 2015). We find that neither 3 *μ*g nor 100 *μ*g of MSS induce an exaggerated increase in Rrs compared with the controls. Indeed, the response observed in mice exposed to MSS is similar to mice exposed to the negative control PBS, and in the area of 200% increase in Rrs compared with the baseline (Fig. 2D). Taking into account the absence of AHR, in addition to the weak presence of CD4/IL‐13^+^ cells at 3 *μ*g of MSS and the absence of IgE, these results indicate that MSS does not induce a type I hypersensitivity allergic reaction in the lung.

**Figure 3:**
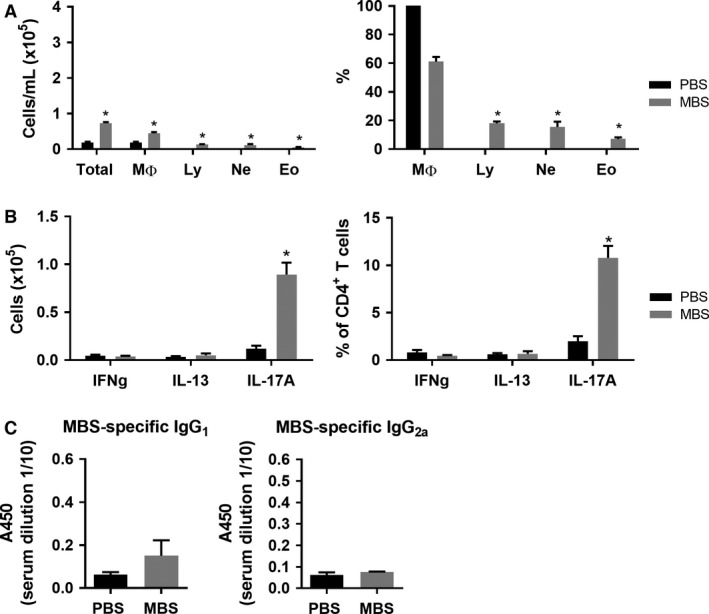
MBS induces a weak T_H_17 immune lung response. (A) Severity of the inflammatory lung response after 6.25 *μ*g MBS exposure was measured using total broncho‐alveolar lavage (BAL) count and differential count. (B) Polarity of the effector response evaluated as number and the % of CD4^+^ cells expressing IFNg, IL‐13 or IL‐17A. (C) MBS‐specific IgG_1_ and IgG_2a_ production was measured from serum using ELISA. Results are representative of at least three separate experiments; *n* = 3–6 mice/group. * = *P* ˂ 0.05.

To test whether other methanogens found in bioaerosols have the potential to induce a type IV hypersensitivity response, we exposed mice to MBS crude extract, which has also been shown to cause an immune response in the lung (Blais‐Lecours et al. 2011). As described before (Blais‐Lecours et al. 2011), BAL counts from mice exposed to 6.25 *μ*g of MBS showed a weaker inflammatory response than 3 *μ*g MSS and this was characterized by fewer granulocytes (Fig. 3A). When looking at the polarity of the effector response, we found that MBS also induces mainly CD4/IL‐17A^+^ cells (Fig. 3B). Furthermore, neither IgG_1_ nor IgG_2a_ production could be observed (Fig. 3C). Low‐dose exposure to MBS therefore induces a T_H_17 response, highlighting its potential to induce a type IV hypersensitivity response, although the overall inflammatory response is weaker than MSS and with less inflammatory cells in the lung.

**Figure 4:**
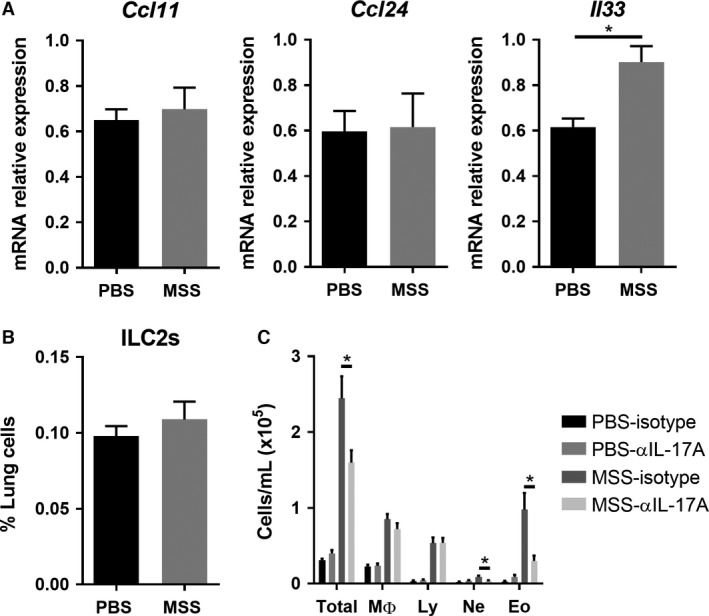
IL‐17A blockade leads to reduced total inflammation and eosinophil influx in the airways. (A) Expression of *Ccl11*,* Ccl24*, and *Il33* measured by qRT‐PCR. (B) The % of ILC2s (Lineage^−^ CD45^+^Sca1^+^ CD90.2^+^ CD25^+^ ST2^+^) in lung of mice exposed to MSS compared with PBS. (C) The severity of the inflammatory lung response in mice that received isotype or anti‐IL‐17A 1 h before each exposure to 3 *μ*g MSS was verified using broncho‐alveolar lavage (BAL) count. Results were pooled from two experiments; *n* = 6–12 mice/group. * = *P* ˂ 0.05.

Eosinophils are present in response to low doses of MSS and the presence of eosinophils in the lung after exposure to non‐infectious agents is anticipated to be associated to allergic, asthma‐like symptoms. Importantly, eosinophils can also contribute to resolution processes in asthma (Takeda et al. 2015), but their significance and role in MSS‐induced airway hypersensitivity remains misunderstood. Therefore, we set out to further evaluate the importance of this cell population in the response to MSS crude extract exposure. However, we found that low‐dose exposure to MSS induces only a weak CD4/IL‐13^+^ response and recruitment of eosinophils (Nakajima et al. 1992; Pope et al. 2001), and no devolopment of AHR, a response associated with the presence of eosinophils (Cockcroft and Davis 2006). Accordingly, we examined the functional significance of eosinophils in the airway inflammatory response to MSS, and on their potential harmful impact in disease. We first assessed the activation of eosinophil recruitment pathways in response to MSS. We analyzed eotaxins and IL‐33 production, which are well‐known chemokines involved in eosinophil recruitment. qRT‐PCR analyses revealed no increase in eotaxins *Ccl11* (Eotaxin‐1) and *Ccl24* (Eotaxin‐2) following MSS crude extract exposure, but an increase in *Il33* mRNA expression (Fig. 4A). IL‐33 is a potent activator of lung ILC2s (Neill et al. 2010), which, in turn, recruit eosinophils in allergy development (Gold et al. 2014). To assess whether MSS‐induced expression of IL‐33 promotes the expansion of lung ILC2s, we quantified lung ILC2s following antigen challenge by flow cytometry (Lineage^−^CD45^+^Sca1^+^CD90.2^+^CD25^+^ST2^+^). The frequency of lung ILC2s was unchanged following exposure to MSS (Fig. 4B). Thus, these results suggest that MSS‐induced eosinophil recruitment to the lungs is not induced by type I hypersensitivity mechanisms.

One of the major cytokine involved in type IV hypersensitivity responses, IL‐17A, is recognized for its role in neutrophil recruitment (Hellings et al. 2003) and stromal cell activation (Molet et al. 2001; Hasan et al. 2013), which can lead to development of fibrosis, such as in HP (Hasan et al. 2013). However, IL‐17A also plays a role in eosinophil recruitment to the lung (Schnyder‐Candrian et al. 2006; Murdock et al. 2012). Thus, we verified whether this cytokine is involved in eosinophil accumulation in response to MSS. To do so, mice were given 50 *μ*g i.p. of anti‐IL‐17A antibodies 1 h prior to 3 *μ*g MSS exposure, and the airway inflammatory response was evaluated. We find that blockade of IL‐17A reduced the total inflammatory response, characterized by fewer numbers of granulocytes, compared with the mice that received the isotype (Fig. 4C). Importantly, the quantity of eosinophils was greatly decreased (Fig. 4C). This suggests that eosinophils accumulate in the lung in response to MSS through type IV hypersensitivity mechanisms, including IL‐17A production.

**Figure 5:**
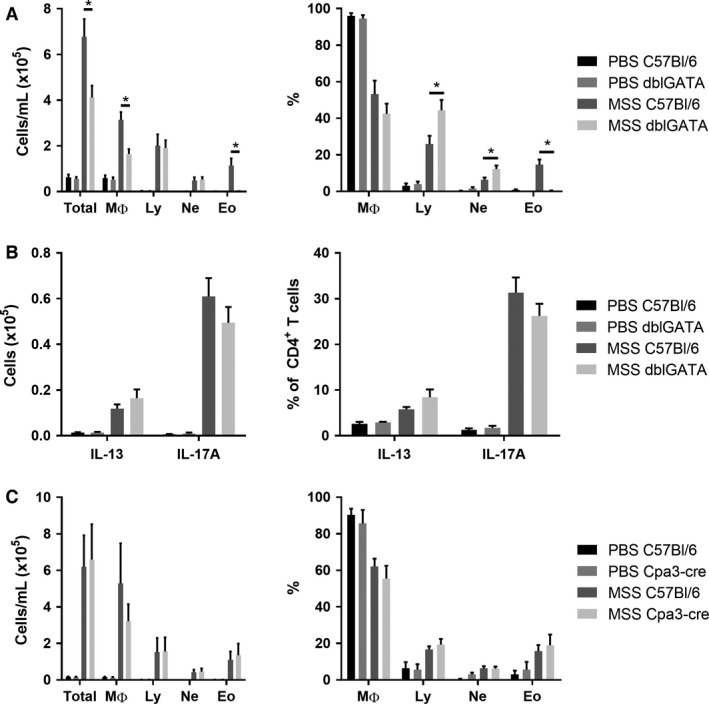
Eosinophils and mast cells are not essential for the development of MSS‐induced airway inflammation. (A) The inflammatory lung response was verified in ΔdblGATA mice after exposure to 3 *μ*g MSS using broncho‐alveolar lavage (BAL) count and differentials. (B) Polarity of the effector response evaluated as number and the % of CD4^+^ cells expressing IFNg, IL‐13 or IL‐17A. (C) Using BAL count, the inflammatory lung response was verified in Cpa3‐cre mice after exposure to 3 *μ*g MSS. Results were pooled from two experiments; *n* = 6–12 mice/group. * = *P* ˂ 0.05.

Using eosinophil‐deficient mice (ΔdblGATA mice), we then set out to directly verify the importance of eosinophils in the lung. As shown by BAL counts, total cells are decreased in ΔdblGATA mice in response to MSS, which is, in large part, a reflection of the lack of eosinophils in these mice (Fig. 5A). However, we also observed a reduced number of macrophages in these mice (Fig. 5A). Furthermore, the polarity of the effector response was not affected by the lack of eosinophils. Indeed, the cell numbers and the % of CD4/IL‐13^+^ and of CD4/IL‐17^+^ cells were similar between ΔdblGATA and WT mice after MSS exposure (Fig. 5B). Thus, the data indicate that eosinophils are dispensable for the development of the type IV hypersensitivity response to MSS. Nevertheless, the decrease in macrophages observed in eosinophil‐deficient mice suggests a role in damage induction for eosinophils in response to MSS, which contributes to the inflammatory response.

Another cell type linked to the T_H_2 response and to the presence of eosinophils is mast cells (Hammad and Lambrecht 2015). As mentioned, MSS exposure induces an increased expression of the eosinophils‐recruiting cytokine *Il33*, which is also a potent activator of mast cells (Hsu et al. 2010). We thus wondered whether mast cells, just like eosinophils, could contribute to the inflammatory response without participating in its development. We verified the importance of mast cells after exposure to MSS by using mast cell‐deficient mice (Cpa3‐Cre mice). BAL counts showed that the lack of mast cells does not affect the severity of the response or the type of cells recruited to the lung (Fig. 5C). Therefore, mast cells are not necessary for the type IV hypersensitivity response to MSS.

## Discussion

The pathologic role of microbial antigens in occupational respiratory disease is poorly understood, in major part because of the complex nature of bioaerosols. With the advent of more precise genomic tools, characterization of bioaerosols revealed a surprisingly, high density of microorganisms, including archaeal species in agricultural buildings (Nehme et al. 2009; Blais‐Lecours et al. 2012; Just et al. 2013). However, the consequences of these bioaerosol components on the health of exposed‐workers remain largely unknown. In this study, we show that the airway exposure to MSS crude extract, an agricultural bioaerosol, induces a type IV hypersensitivity lung mucosal immune response in mice, thus confirming its harmful potential for humans.

Characterization of the response in WT mice demonstrates that chronic MSS exposure leads to a strong lymphocytosis, where naïve CD4^+^ T cells are skewed towards T_H_17 cells, and to the expression of *Il17a* in the lung. Using anti‐IL‐17A antibodies, we confirmed a crucial role for IL‐17A in the development of the inflammatory response to MSS, as these led to a significant decrease in the total inflammation in the lung. The results are consistent with a cell‐mediated, type IV hypersensitivity response to MSS. Previously, Blais‐Lecours et al. described the formation of tertiary lymphoid tissues in the lung in response to MSS, which we also observed (data not shown), as a characteristic of a type IV lung hypersensitivity response (Suda et al. 1999). Another possible component of a type IV hypersensitivity response is a strong cytotoxic activity of CD8 T cells immediately following antigen exposure. While exposure to MSS crude extract induces only a mild recruitment of CD8^+^ T cells in the BAL of mice 4 days after the last exposure (Blais‐Lecours et al. 2011), suggesting that they do not play an important role at this time point, their rapid recruitment following MSS exposure (less than 24 h) has not yet been assessed and requires further investigation.

MSS also induces isotype class switching to IgG_1_ and IgG_2a_, which result from a strong IL‐17A production (Mitsdoerffer et al. 2010). As described in the introduction, high antibody titers can lead to type II or III hypersensitivity. More precisely, in type II responses, antibody ligation by host cells activates the complement cascade, inducing apoptosis (Descotes and Choquet‐Kastylevsky 2001; Warrington et al. 2011). In type III responses, excess immune complexes precipitate at the site of inflammation, leading to activation of the immune response (Descotes and Choquet‐Kastylevsky 2001; Warrington et al. 2011). Whether MSS‐specific IgGs induce a type II or a type III response in the lung requires further investigation.

At low quantities, MBS crude extract polarized naïve T cells towards T_H_17 differentiation. However, there was no increase of IgG_1_ or IgG_2a_, as this archaeon is less immunogenic than MSS (Blais‐Lecours et al. 2011). Thus, whether MBS crude extract also induces a type IV hypersensitivity response remains to be determined. Experiments using a higher quantity of MBS might answer this question. Nevertheless, this result suggests that, in general, methanogens are able to induce a T_H_17 polarized mucosal response, which leads to a type IV hypersensitivity response depending on the immunogenic potential of the archaeon. The immunogenicity of other methanogen species and whether they also induce a strong type IV lung response by an increase of T_H_17 CD4^+^ T cells may provide further insights into this hypothesis. The non‐endogenous methanogen species *Methanobrevibacter woesei*, which is found in poultry farms, environments high in bioaerosols that cause airway diseases and symptoms in workers (Donham et al. 2000; Just et al. 2013), could be used to test this intriguing hypothesis.

We also found a weak polarization towards T_H_2 responses at the lowest dose of MSS, which is in accordance with the high quantity of eosinophils observed in the BAL at this dose. However, this weak T_H_2 response was not accompanied with IgE isotype switching and AHR, the hallmarks of lung T_H_2 allergic responses (Holgate 2012). Thus, our data argue that MSS fails to generate a full type I hypersensitivity response like that found in asthma.

Although our results suggest that MSS induces a type IV, and not a type I, hypersensitivity response, the important recruitment of eosinophils in response to MSS crude extract exposure is intriguing, and raises questions towards their role in this specific immune response. Further investigation revealed no increase in eotaxins or ILC2s after exposure to MSS, suggesting that IL‐17A may be at cause for eosinophil trafficking. Indeed, studies demonstrated that IL‐17A is important in the initiation of allergic responses, as its absence leads to a reduction of eosinophil recruiting and activating cytokines (Nakae et al. 2002; Song et al. 2008). Accordingly, in our model, antibody blockade of IL‐17A reduced the eosinophil numbers in the airways, suggesting that IL‐17A production induced by MSS could partially explain the recruitment of eosinophils in the lung. Furthermore, we found an increase in *Il33* expression in MSS‐exposed WT mice, which could partially explain the recruitment of eosinophils, as it is a cytokine known to activate and recruit eosinophils (Pecaric‐Petkovic et al. 2009; Rankin et al. 2010). Surprisingly, when looking at the importance of these cells in the inflammatory response using ΔdblGATA mice, results show that eosinophils are not necessary for the development of the inflammatory response to MSS. Rather, they likely contribute to exacerbating this response via the induction of damage in the lung (Frigas et al. 1980; Motojima et al. 1989; Flood‐Page et al. 2003), which is supported by the reduced number of macrophages in eosinophil‐deficient mice. Thus, in addition to the damages induced directly by the hypersensitivity type IV response, exposure to MSS can cause lung tissue damage by recruiting eosinophils.

Our results using mast cell‐deficient mice lead to the conclusion that these cells do not play a role in the hypersensitivity response induced by MSS. On another note, we found an increase in *Il33* expression and mast cells are producers of IL‐33, although the major IL‐33‐producing cells in the lung are epithelial cells (Hsu et al. 2010; Hardman et al. 2013). However, as we found no differences in the severity of the inflammatory response in mast cell‐deficient mice, mast cells are not likely to be a major source of IL‐33 in the response to MSS. This raises the possibility that MSS is able to stimulate epithelial cells to produce IL‐33. The mechanisms by which MSS could activate epithelial cells remain to be identified, as it was shown to be incapable of inducing an IL‐8 release in human epithelial cell lines transformed with different TLRs and NLRs (Bang et al. 2014).

High levels of endotoxins are found in bioaerosols, and workers are likely to be co‐exposed to endotoxins and MSS simultaneously (Clark et al. 1983; Nehme et al. 2009). Some endotoxin was found in the archaeal preparations lot used in this study (see exact numbers in methods), which is a different lot from the Blais‐Lecours study (2011). Importantly, the presence of LPS in the MSS preparation did not influence the inflammatory response to MSS. Indeed, our BAL content results are fully in line with Blais‐Lecours et al.'s (2011) previous study. Therefore, we postulate that the combined exposure to endotoxins and MSS does not deviate from the type IV hypersensitivity response observed, which is highly relevant for workers as they are exposed to high levels of endotoxins bioaerosols (Clark et al. 1983).

In conclusion, our data demonstrate that chronic exposure to the archaeon MSS induces a lung mucosal response typical of a type IV hypersensitivity response, but not a type I, classical allergic response. Whether this type of response is also induced by endogenous methanogens in the gut and mouth remains to be confirmed; however, our results suggest that MSS could participate in gut diseases such as IBD, where they were found in higher quantity (Blais‐Lecours et al. 2014). Importantly, the effect of other methanogens, such as the human non‐endogenous species *Methanobrevibacter woesi* found in poultry farms (Just et al. 2013), should be evaluated, as workers from these environments are also prone to develop airway inflammatory diseases (Donham et al. 2000). Finally, our study demonstrates that the type IV hypersensitivity response elicited by MSS could participate in the development of lung hypersensitivity diseases in workers exposed to MSS bioaerosols.

## Conflict of Interest

The authors declare that they have no competing interests.
